# A game theoretical model for the stimulation of public cooperation in environmental collaborative governance

**DOI:** 10.1098/rsos.221148

**Published:** 2022-11-09

**Authors:** Yinhai Fang, Matjaž Perc, Hui Zhang

**Affiliations:** ^1^ College of Economics and Management, Nanjing Forestry University, Nanjing 210037, People's Republic of China; ^2^ Faculty of Natural Sciences and Mathematics, University of Maribor, Koroška cesta 160, 2000 Maribor, Slovenia; ^3^ Department of Medical Research, China Medical University Hospital, China Medical University, Taichung 404332, Taiwan; ^4^ Alma Mater Europaea, Slovenska ulica 17, 2000 Maribor, Slovenia; ^5^ Complexity Science Hub Vienna, Josefstädterstraße 39, 1080 Vienna, Austria

**Keywords:** game theory, human behaviour, cooperation, mathematical model, common goods

## Abstract

Digital technologies provide a convenient way for the public to participate in environmental governance. Therefore, by means of a two-stage evolutionary model, a new mechanism for promoting public cooperation is proposed to accomplish environmental collaborative governance. Interactive effects of government–enterprise environmental governance are firstly explored, which is the external atmosphere for public behaviour. Second, the evolutionary dynamics of public behaviour is analysed to reveal the internal mechanism of the emergence of public cooperation in environmental collaborative governance projects. Simulations reveal that the interaction of resource elements between government and enterprise is an important basis for environmental governance performance, and that governments can improve this as well as public cooperation by increasing the marginal governance propensity. Similarly, an increase in the government's fixed expenditure item of environmental governance can also significantly improve government–enterprise performance and public cooperation. And finally, the effect of government's marginal incentive propensity on public environmental governance is moderated by enterprises' marginal environmental governance propensity, so that simply increasing the government's marginal incentive propensity cannot improve the evolutionary stable state of public behaviour under the scenario where enterprises’ marginal environmental governance propensity is low.

## 1. Introduction

Ecological environment is a prerequisite for ensuring high-quality development of economy and society [1,2]. However, the sustainability and effectiveness of environmental governance paradigm is hard achieve without public participation in social-ecological systems [3–8], due to residents being concerned more about pollution and environmental issues while reaching middle-income status, reducing the cost of environmental governance and significantly improving the effectiveness of green governance [9–11]. Traditional vertical environmental governance dominated by the central-local governments is prone to defects such as information asymmetry [12,13] and weak supervision [14]. The roles of both public behavioural participation and policy participation in environmental governance are alternatives to each other [15], and environmental governance shifts away from a centralized system to a multi-actor system underpinned by public participation in decision-making [6,16].

In China, the central government has developed a series of policies and measures to guide and stimulate public participation in environmental governance. For example, ‘Guiding Opinions on Building a Modern Environmental Governance System (MEGS)’ was issued in March 2020. However, when local governments or pollution enterprises fail to perform their duties responsibly, the public lacks adequate legal and administrative remedies [17]. Therefore, it is difficult to produce the collaborative effect of public participation without constructive measures in environmental collaborative governance.

From the perspective of public participation, there is considerable scope for ambitious environmental policies, even under adverse economic conditions [18]. However, public cooperative behaviour motivation is a relatively new concept in China's environmental policy design, and it has been rarely studied [5,19]. The core question of this research is how to motivate the public to participate more effectively in the multi-agent environmental collaborative governance. The interactive nature of environmental governance among stakeholders makes the group behaviour evolution dynamics model a suitable tool for studying public participation in the context of collaborative environmental governance. Compared with traditional game theory, it combines bounded rationality, autonomous learning and strategy dynamics of decision makers. Initially, the evolutionary dynamics of group behaviour were used by statistical physicists for the emergence mechanism of group cooperative behaviour [20–22], and later gradually extended to many areas of social management [23–25].

This paper seeks to move a step forward for public participation in environmental collaborative governance by integrating economics, behaviour and environmental governance into a coherent framework through evolutionary dynamics model. The novelty and contribution of this study are presented as follows: first, it provides an understanding of the importance of the interaction between local governments and pollution enterprises in environmental governance system. The government–enterprise interaction reveals how marginal governance propensity, marginal incentive propensity and fixed expenditure items of environmental governance affect their environmental performance.

Second, this research mainly focuses on the behaviour evolutionary dynamics of interactive game with multi-agent and multi-stage [20,26] and promotes the emergence of public cooperative behaviour in the issue of environmental collaborative governance. Specifically, this study attempts to answer three questions: how do local governments and pollution enterprises actively interact to create an external atmosphere for public participation? What are the conditions for the emergence of cooperative behaviour of public participation in environmental governance? How the enthusiasm of the public to participate in environmental governance evolves over time and its spatial distribution characteristics. Although several studies have conducted important research on public participation in environmental governance [5,19,27], the above issues are not at the core of their research design. Our research question has not been fully explored.

Third, our research method is a policy simulation of public participation in environmental governance based on behavioural evolutionary dynamics, which is different from other studies. This application of behavioural evolution dynamics is very novel and can better reveal how to stimulate the enthusiasm of public participation, thereby improving the overall efficiency of environmental governance. Simultaneous effects of multiple policy instruments were systematically examined in policy simulations, whereas previous similar analyses often conducted independent sensitivity analyses for individual parameters. In addition, the research design of the evolution dynamics of public environmental governance behaviour in this paper is not designed to prove the robustness of theoretical propositions commonly found in the existing literature [28,29], so it is more policy-oriented predictability of effects.

Our evolutionary dynamics simulation results show that stimulating public participation in environmental governance and realizing the emergence of cooperative behaviours can be done from different perspectives. From the macro-perspective, the cooperative behaviour of public participation in environmental governance is seriously affected by the marginal propensity, incentive propensity and fixed expenditure of the government and enterprises, which determine the macro-environmental governance performance. Then, from the micro-perspective, based on the analysis of the influencing factors of behavioural decision-making of public participation in environmental collaborative governance, it is revealed that the cooperative behaviour of public participation in environmental governance diffuses among the population, which can better play the role of macro-environmental governance policies in regulating public behaviour.

The remaining paper is organized as follows. Section 2 is the literature review; §3 briefly explains the theoretical mechanisms of environmental governance combining government, enterprises and publics, and puts forward a two-stage model of environmental collaborative governance; §4 shows simulation results including the interaction of environmental governance between the government and enterprises, and evolution dynamics of public behaviour in environmental governance; Lastly, we end the paper with some discussions in §5.

## 2. Literature review

This research mainly involves literature in three areas. The first area is about public participation in environmental governance. As the topic of extensive research in terms of environmental public goods supply, public participation can be traced back to the classical fiscal federalism theory [30]. Studies in this category have paid enough attention to the strategic interactions in environmental policy triggered by the devolution of power from the central to local governments [31,32]. For example, the relevant external factors may modify competitiveness and exclusiveness of environmental public goods [33]. When the positives outweigh the negative externalities, the publics' willingness to govern the environment will decline and their behaviour will evolve into a free-rider phenomenon of environmental governance [34]. However, public participation in environmental governance is gradually institutionalized. For example, the central government guarantees public participation through a system that compels local governments to disclose the environmental information they hold [35], and the number of environmental NGOs in China has been growing rapidly for a long time [6,36].

The second area mainly involves the positive effects of public participation on environmental governance. Environmental impact assessment (EIA) is widely adopted by western countries as a policy tool [37,38], and its adoption can create possibilities for public participation during early environmental protection and planning stages [38]. Meanwhile, as a basic part of EIA, public participation is beneficial to local governments to publish environmental monitoring data in a timely and transparent manner [39]. The mutual benefit of public participation and other stakeholders in environmental governance is another focus of this literature [40–42]. For example, strong public support can ensure that environmental policies achieve emission reduction targets under weak environmental regulation [43]. As a wide-scale system of environmental information disclosure, public participation is expected theoretically to be the link between government-led vertical governance [44–46] and market-oriented horizontal governance [28,47]. However, the complementary effect is influenced by a wide range of factors, ranging from the promotion of information exchange mechanisms [48,49] to the optimization of governance strategies [50,51]. Public appeals for environmental issues can significantly enhance government's control over environmental pollution [52], central government's oversight of local governments [53] and enterprises‘ innovation behaviour [54]. The institutional design of governance mechanisms is important for building relationships and shaping policy outcomes [55], e.g. objectives and designs of public participation that are communicated explicitly, and discussed by participants, can increase the chances of achieving environmental governance objectives [56]. However, relying solely on strong institutional design for public participation cannot guarantee that the publics can achieve substantial autonomy from the power structure in actual participatory governance [57].

The third area concerns the application of behaviour evolutionary dynamics to public participation in environmental governance. The traditional evolutionary game method is suitable for quantitative analysis of the iterations and interactions between central environmental protection department (CEPD), local environmental protection department (LEPD) and carbon emission enterprise (CEE) in environmental governance research, and reveals their evolutionary paths to provide reference reform suggestions for environmental governance [58]. More participatory approaches to tackling environmental challenges have the capacity to reduce conflict among different stakeholders and publics [59–61]. Environmental governance performance of the government, enterprises and publics are analysed based on the evolutionary game framework [5], and it turns out that enthusiasm of public participation is closely related to participation costs and psychological benefits. Considering that the public participation in environmental collaborative governance is essentially a complex and systematic decision-making problem composed of government, enterprises and publics, the evolutionary dynamics of cooperation [20,21,62,63] may provide a potential analytical tool for collaborative environmental governance. Behavioural evolutionary dynamics not only considers the irrational factors of public participation [58], but also involves reputation [64,65], conformity [66], individual diversity [67], social network relationships [68], etc. In addition, multi-agent simulation can better deal with the complexity and dynamics of environmental problems, attracting many scholars to conduct environmental science research based on complex system dynamics simulation [69–72].

In previous literature, scholars have conducted in-depth analyses of the positive effects of public participation in environmental governance and key factors affecting its improvement, which are important references for this study. Unfortunately, there are still some shortcomings in prior research. First, while some previous studies focused on the impact of public participation in environmental governance (e.g. environmental democracy, authoritarianism, investment and supervision), the social network of the publics involved in environmental governance has not been fully revealed, and the incentive mechanisms of public environmental behaviour in multi-organism collaborative governance need to be further explored. This study establishes a two-stage behavioural evolutionary dynamics model to explore the micro-mechanisms behind the collaborative environmental governance of government, enterprises and publics, and sheds light on how public participation can render environmental governance.

## 3. Model

Relying solely on local governments is difficult to meet the needs of a fully comfortable ecological environment, and it is necessary to establish an environmental governance system led by the government, responded to by enterprises and participated in by the public. In the first phase, the rational and effective allocation of ecological environmental resources must rely on optimizing the interaction mechanisms between government and enterprise. Government, as the leading force in environmental governance, plays an important role in environmental decision-making, implementation and supervision, while enterprises are the main practitioners of environmental protection and restoration. Efficient interaction between them is a core component of environmental construction. Therefore, the first stage focuses on analysing the interaction between government and enterprise, based on which an environmental governance model of government–enterprise is constructed. The second stage considers that the macro-level environmental governance performance can reflect the overall ecological environment, which has some influence on the social public participation behaviour in environmental governance. Specifically, the public involved in eco-environmental governance mainly refers to all those who are interested in or affected by environmental decision-making, that is, they enjoy the right to access environmental information, have the right to participate equally in all decision-making involving environmental interests in accordance with relevant channels and procedures, and therefore need to assume the responsibility and obligation to perform environmental protection. In the second stage, taking the government–enterprise environmental governance performance in the first stage as an important factor, the evolutionary dynamics model of public behaviour in environmental collaborative governance was constructed.

### 3.1. Interaction model of government–enterprise environmental governance

Direct interaction of resource elements between government and enterprises will occur in the process of environmental governance. Specifically, the government's transfer payments to enterprises’ environmental governance efforts can be regarded as the component of enterprises' environmental governance performance. For example, the government's financial incentives and tax breaks for the upgrading of enterprises' green technology are conductive to increasing the willingness and efficiency of enterprises to improve environmental governance, which in turn will be reflected in their environmental governance performance. Meanwhile, the enterprises’ efforts to cooperate with the government to accomplish the environmental governance tasks can be regarded as the component of government's environmental performance. Assuming that governments and enterprises still have the possibility of improving environmental performance at the current economic and technological level, it depends not only on the externalities mentioned above, but also on the governors' own behaviour, such as marginal environmental governance propensity, environmental governance fixed expenditure items, etc. Therefore, the constant equation for the government's environmental performance (*Y_G_*) is defined as

3.1 $$Y_{G,t+1}=\beta_{G}Y_{G,t}+a_{G}+h_{G,t}-m_{G}Y_{G,t},$$

where $Y_{G,t}$ and $Y_{G,t+1}$ denote government's environmental governance performance at time of *t* and t+1, respectively; $\beta_{G}$ is the government's marginal environmental governance propensity; $a_{G}$ is the government's environmental governance fixed expenditure item, specifically including the costs of environmental infrastructure construction, pollution regulation, remediation and punishment determined by the government's annual budget; $h_{G,t}$ is the government's environmental governance-related benefits from enterprises at time of *t*, such as the benefits of providing non-public support services to enterprises; $m_{G}$ is the government's marginal incentive propensity for the enterprise environmental governance.

Similarly, the constant benefit equation for enterprises’ environmental governance performance is defined as

3.2 $$Y_{E,t+1}=\beta_{E}Y_{E,t}+a_{E}+h_{E,t}-m_{E}Y_{E,t},$$

where $Y_{E,t}$ and $Y_{E,t+1}$ are the performance of enterprise's environmental governance at time of *t* and t+1, respectively; $\beta_{E}$ is the enterprise's marginal environmental governance propensity; $a_{E}$ is the enterprise's environmental governance fixed expenditure item, for example, the enterprise's procurement of relevant environmental protection equipment, operation of pollution-free equipment for waste gas, sewage and waste residue, design of new production processes or use of new raw materials with less polluting emissions; $h_{E,t}$ is the enterprise's environmental governance resource element from government at time *t*, including non-profit professional guidance, tax incentives and special financial support for enterprises' environmental pollution control measures; *m_E_* is the marginal demand propensity from enterprises for government environmental governance paid services, for example, the cost of government-led third-party professional organizations to provide enterprises with experience and technical support for the whole process of environmental governance.

The interaction of resource elements in the process of collaborative environmental governance between the government and enterprises is conducive to their respective environmental governance performance. Among them, the environmental governance-related benefits obtained by the government from enterprises (*h_G_*) depend to a certain extent on the marginal demand propensity from enterprises for government environmental governance paid services and enterprise's environmental governance performance; the environmental governance-related benefits obtained by enterprises from the government (*h_E_*) depend to a certain extent on the government's marginal incentive propensity for enterprise environmental governance and government's environmental governance performance. It is assumed that *h_G_* and *h_E_* at time *t* satisfy

3.3 $$h_{G,t}=m_{E}Y_{E,t}$$

and

3.4 $$h_{E,t}=m_{G}Y_{G,t}.$$

Substituting the above results into equations (3.1) and (3.2), respectively, the interactive model of government–enterprise environmental governance is as follows:

3.5 $$Y_{G,t+1}=\beta_{G}Y_{G,t}+a_{G}+m_{E}Y_{E,t}-m_{G}Y_{G,t}$$

and

3.6 $$Y_{E,t+1}=\beta_{E}Y_{E,t}+a_{E}+m_{G}Y_{G,t}-m_{E}Y_{E,t}.$$

Therefore, the equilibrium of the environmental performance benefits of the government and enterprises is

3.7 $$\begin{array}{rl} Y_{G}^{*}= & \left(\frac{K_{G}^{I}}{1-K_{G}^{I}K_{E}^{I}m_{G}m_{E}}\right)(a_{G}+m_{E}K_{E}^{I}a_{E})\\ Y_{E}^{*}= & \left(\frac{K_{E}^{I}}{1-K_{E}^{I}K_{G}^{I}m_{E}m_{G}}\right)(a_{E}+m_{G}K_{G}^{I}a_{G}) \end{array}\}.$$

Among them, $K_{G}^{I}=1/(1-\beta_{G}+m_{G})$ and $K_{E}^{I}=1/(1-\beta_{E}+m_{E})$ are the open environmental governance multipliers of the government and enterprises, respectively, which do not involve the interdependence of the two types of subjects also referred to as non-dependency multipliers. However, it is often the case in environmental governance practice that the environmental governance behaviour of government or enterprises affects the environmental governance performance of other agents through certain paths, i.e. $K_{G}^{I}/(1-K_{G}^{I}K_{E}^{I}m_{G}m_{E})$ and $K_{E}^{I}/(1-K_{E}^{I}K_{G}^{I}m_{E}m_{G})$ are the government environmental governance dependency multiplier $\left(K_{G}^{D}\right)$ and the enterprise environmental governance dependency multiplier $\left(K_{E}^{D}\right)$ under the interaction, respectively. Since the denominators of $K_{G}^{D}$ and $K_{E}^{D}$ are positive numbers less than 1, the environmental governance dependency multipliers are larger than the non-dependency multipliers, and the agents with larger non-dependency multipliers correspond to larger dependency multipliers. According to $Y_{G}^{*}$, it is known that the marginal demand propensity of enterprises for paid services, the non-dependency multiplier and the fixed expenditure item of environmental governance will affect the equilibrium level of government environmental governance performance. Further analysis reveals that $Y_{G}^{*}=K_{G}^{I}a_{G}<\;[K_{G}^{I}/(1-K_{G}^{I}K_{E}^{I}m_{G}m_{E})](a_{G}+m_{E}K_{E}^{I}a_{E})$ if there is only government environmental governance, implying that the interaction between government and enterprises has a positive meaning.

### 3.2. Evolutionary dynamics model of government–enterprise–public environmental governance

**Figure 1.**
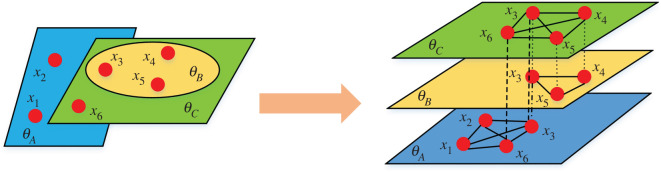
The proposed micro-environmental public goods model from the perspective of multi-layer network in this paper. $\theta_{A}$, $\theta_{B}$ and $\theta_{C}$ are the micro-environmental public goods, respectively. The lines between different layers mean that nodes are the same individuals who participate in different micro-environmental public goods, e.g. dash-dotted lines represent simultaneous participation in $\theta_{A}$ and $\theta_{B}$; dashed lines represent simultaneous participation in $\theta_{A}$ and $\theta_{C}$; dotted lines represent simultaneous participation in $\theta_{B}$ and $\theta_{C}$. The solid lines within the same network layer indicate that the individuals participate together in a specific environmental public goods game.

The value attributes of environmental public goods are mainly determined by the environmental governance performance of the government, enterprises and publics, and are usually indivisible, non-exclusive and non-competitive within the same region. With the general increase in public education and the widespread use of information technology in environmental protection publicity and regulation, the share of publics' environmental governance performance in the value of environmental public goods will continue to increase. Unlike behaviours of the government and enterprises under the constraints of certain laws and regulations, the environmental governance activities of publics are more reflective of public goods and autonomy. In addition, the performance of individual environmental behaviour directly affects the value of environmental public goods in their communities and units on a microscopic scale. Therefore, regional ecological environmental public goods can be further divided into many micro-environmental public goods (*θ*) based on the communities in which the publics live. Considering that multi-layer network has obvious advantages in expressing the behavioural characteristics of multiple subjects in different networks, a micro-environmental public goods model is constructed based on the multi-layer network perspective. For example, as shown in figure 1, individual $x_{3}$ maintains and shares $\theta_{A}$ with $x_{1}$, $x_{2}$ and $x_{6}$ in community *A*, $\theta_{B}$ with $x_{4}$ and $x_{5}$ in community *B*, and $\theta_{C}$ with $x_{4}$, $x_{5}$ and $x_{6}$ in community *C*. Thus, the set of micro-environmental public goods in which individual $x_{3}$ participates is $\Theta_{x_{3}}=\{\theta_{A},\theta_{B},\theta_{C}\}$. Similarly, the set of micro-environmental public goods in which individual $x_{4}$ participates is $\Theta_{x_{4}}=\{\theta_{B},\theta_{C}\}$. Finally, the set of micro-environmental public goods in which all individuals in the community participate is $\Theta =\cup \Theta_{x_{i}}$.

Differences in individual living status and environmental cognition during the consumption of micro-environmental public goods lead to differences in their environmental governance behaviours, which in turn induce the ‘free-rider’ problem of environmental public goods governance. When individuals are exposed to environmental pollution, there are usually two strategies of ‘no resistance’ and ‘resistance’, but mainly in the case of major environmental pollution outbreaks. For general environmental governance issues, publics' behaviour is influenced by the government's emphasis on environmental governance and will weigh their own compensation for participating in environmental protection activities, usually showing indifference and enthusiasm. The former refers to publics ignoring environmental issues and not participating in direct environmental governance activities, while the latter means active participation in environmental governance activities. To facilitate the modelling analysis, the behaviour of publics in collaborative governance is abstracted into cooperation and defection. The cost borne by individual $x_{i}$ for cooperating in the governance of micro-environmental public goods (*θ_j_*) is $\Omega_{\theta_{j}}$, including indirect and direct cost. Accordingly, the benefits obtained by individual $x_{i}$ in micro-environmental public goods through cooperation and defection are

3.8 $$P_{C,x_{i}}=\underset{\theta_{j}\in \Theta_{x_{i}}}{\sum }\left[\frac{\gamma (N_{C,\theta_{j}}\Omega_{\theta_{j}})}{N_{\theta_{j}}}-\Omega_{\theta_{j}}\right]$$

and

3.9 $$P_{D,x_{i}}=\underset{\theta_{j}\in \Theta_{x_{i}}}{\sum }\frac{\gamma (N_{C,\theta_{j}}\Omega_{\theta_{j}})}{N_{\theta_{j}}},$$

where $N_{\theta_{j}}$ and $N_{C,\theta_{j}}$ are the group size of the micro-environmental public goods ($\theta_{j}$) and the number of individuals who take cooperative behaviour among them, respectively; $\Theta_{x_{i}}$ is the set of micro-public goods in which individual $x_{i}$ is located and *γ* is the amplification coefficient of the environmental performance of individual cooperative behaviour, which reflects the excess performance of the collaborative environmental governance process. Considering that the individual practice of value-rational oriented environmental governance cooperative behaviour depends to some extent on the external overall environmental governance status. Therefore, it is assumed that γ is positively correlated with the level of government–enterprise environmental governance performance. The higher government–enterprise environmental governance performance *Y* ($Y=Y_{G}^{*}+Y_{E}^{*}$) than the eco-vigilance indicator *Y_S_*, the more obvious is the amplification effect of cooperative governance behaviour, e.g. $\gamma =\log_{Y_{S}}Y$ (*Y* *>* *Y_S_*).

Public participation in environmental governance can improve the plight of insufficient supply of environmental public goods and reduce the pressure and crisis of political trust and financial revenue and expenditure of the government. However, environmental collaborative governance by multiple agents is essentially a matter of choice and adjustment based on interest preferences. Publics' behavioural decisions in environmental governance depend on the benefits of governance performance on the one hand and are also influenced by the behaviour of neighbouring agents on the other hand. Specifically, individual *x_i_* selects a neighbour *x_j_* in the same community as the object of emulation, and the probability of successful emulation depends on the difference in environmental governance performance between them. Therefore, the Fermi function [73] can be used to determine the probability of individual environmental governance behaviour transition as follows:

3.10 $$W(s_{x_{i}}\to s_{x_{j}})=\frac{1}{1+\exp\;[(P_{x_{i}}-P_{x_{j}})/K]}.$$

Among them, $s_{x_{i}}$ and $s_{x_{j}}$ are the environmental governance behaviours of $x_{i}$ and $x_{j}$ respectively; $x_{j}$ is the neighbouring subject of *x_i_* and *K* is the noise coefficient, which can be used to regulate the degree of influence of neighbouring subject's environmental governance behaviour on itself. For example, K→0 and K→∞ refer to the complete dependence on the behaviour of neighbouring subject and completely random behaviour, respectively, and K∈(0,1) indicates the uncertainty level of individual behaviour. The noise coefficient was usually taken as *K* = 0.1 in previous studies [74,75]. In the case with the same environmental noise, if $P_{x_{i}}\gg P_{x_{j}}$, then $W(s_{x_{i}}\to s_{x_{j}})\to 0$, which indicates that individual $x_{i}$ will maintain the current behaviour at the next round. Conversely, $W(s_{x_{i}}\to s_{x_{j}})\to 1$ indicates that $x_{i}$ will follow the environmental governance behaviour of $x_{j}$ at the next round.

## 4. Results

### 4.1. Dynamic evolution of government–enterprise environmental governance

**Figure 2.**
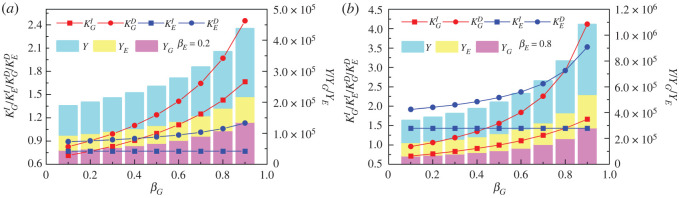
Influence of government marginal governance propensity ($\beta_{G}$) on dependency multipliers ($K_{G}^{D}$ and $\;K_{E}^{D}$), non-dependency multipliers ($K_{G}^{I}$ and $\;K_{E}^{I}$) and environmental governance performance ($Y_{G}$,$\;Y_{E}$ and  Y). (*a*,*b*) correspond to the two scenarios with low ($\beta_{E}=0.2$) and high ($\beta_{E}=0.8$) marginal environmental governance propensity of enterprise, respectively. The red circles and squares stand for government dependency ($K_{G}^{D}$) and non-dependency multipliers ($K_{G}^{I})$. The blue circles and squares stand for enterprise dependency ($K_{E}^{D}$) and non-dependency multipliers ($K_{E}^{I})$. The light magenta, light yellow and light cyan bars stand for environmental governance performance of government, enterprise and the total, respectively. The other parameters are as follows: $m_{G}=m_{E}=0.5$, $a_{G}=a_{E}=40\;000$, K=0.1.

The interaction of environmental governance resources and elements between the government and enterprises is an important basis for generating environmental governance performance. It can be seen from figure 2*a* that the increase in the government's marginal environmental governance propensity will lead to an increase in its own environmental governance dependent multiplier and non-dependent multiplier, and the former will increase more significantly. For example, the increase values of $K_{G}^{D}$ and $K_{G}^{I}$ are 1.07 and 0.66, respectively, when $\beta_{G}$ increases from 0.2 to 0.8. It implies that government can improve performance by increasing marginal governance propensity in the process of environmental governance and create environmental governance spillover benefits by enhancing interaction with enterprises (*Y_E_* improves significantly with the increasing *β_G_*). Environmental governance performance of the government and enterprises is improved respectively, which in turn leads to the improvement of the overall government–enterprise environmental performance. For example, the marginal propensity of government environmental governance increases by 0.4 (i.e. *β_G_* increases from 0.1 to 0.5), and the government–enterprise overall environmental governance performance increases by 33*%* (i.e. the value of *Y* increases from 94 268 to 125 714).

Further, from figure 2*b*, it can be found that the boosting effect of higher marginal propensity of government environmental governance on environmental performance is also influenced by the marginal propensity of enterprise environmental governance. For a given level of the government marginal governance propensity, the higher enterprise marginal governance propensity corresponds to a higher dependency multiplier and environmental performance. In addition, the non-dependency multiplier of government increases while that of enterprises remains unchanged. Moreover, the above positive moderating effect is enhanced by the increase of government marginal governance propensity. Specifically, when $\beta_{G}=0.2$ and *β_E_* increases from 0.2 to 0.8, the corresponding $K_{G}^{D}$, $K_{G}^{I}$, $K_{E}^{D}$, $K_{E}^{I}$ and *Y* increase by 0.16, 0, 1.07, 0.66 and 81 818, respectively; when $\beta_{G}=0.8$ and *β_E_* increases from 0.2 to 0.8, the corresponding $K_{G}^{D}$, $K_{G}^{I}$, $K_{E}^{D}$, $K_{E}^{I}$ and *Y* increase by 0.95, 0, 1.86, 0.66 and 218 181, respectively. Thus, the contribution of enterprise marginal governance propensity increases with the increase of government marginal governance propensity, i.e. there is an interactive synergistic effect of the marginal propensity of government and enterprise. In terms of growth rate, the original *β_G_* increases from 0.1 to 0.5 leading to a 33% increase in the combined government–enterprise environmental performance, and it increases by 155% for the same change in *β_G_* when *β_E_* increases from 0.2 to 0.8.

**Figure 3.**
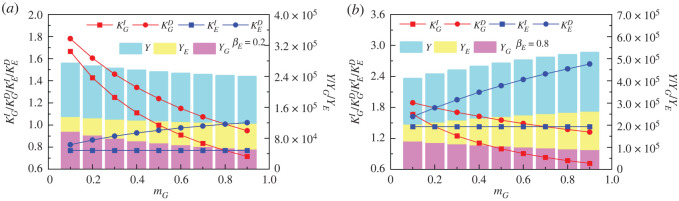
Influence of government marginal incentive propensity (*m_G_*) on dependency multipliers ($K_{G}^{D}$ and $\;K_{E}^{D}$), non-dependency multipliers ($K_{G}^{I}$ and $\;K_{E}^{I}$) and environmental governance performance ($Y_{G}$, $\;Y_{E}$ and *Y*). (*a*,*b*) correspond to the two scenarios with low ($\beta_{E}=0.2$) and high ($\beta_{E}=0.8$) marginal environmental governance propensity of enterprise, respectively. The red circles and squares stand for government dependency $\left(K_{G}^{D}\right)$ and non-dependency multipliers $\left(K_{G}^{I}\right)$. The blue circles and squares stand for enterprise dependency $\left(K_{E}^{D}\right)$ and non-dependency multipliers $\left(K_{E}^{I}\right)$. The light magenta, light yellow and light cyan bars stand for environmental governance performance of government, enterprise and the total, respectively. The other parameters are as follows: $m_{E}=0.5$, $\beta_{G}=0.5$, $a_{G}=a_{E}=40\;000$, K=0.1.

Unlike the marginal governance propensity, the effect of the level of government incentives for enterprise environmental governance on the government–enterprise environmental performance varies considerably across the enterprise marginal governance propensity. As shown in figure 3*a*, government dependency multipliers, non-dependency multipliers and environmental performance decrease gradually with the increase of government's marginal incentive propensity to enterprises. For example, the numerical increments of $K_{G}^{D}$, $K_{G}^{I}$ and $Y_{G}$ are −0.60, −0.66 and −33074.90, respectively, when *m_G_* increases from 0.2 to 0.8. It results in a downward trend in government environmental performance. By contrast, $K_{E}^{D}$ and $Y_{E}$ increase slightly as $m_{G}$ increases (the numerical increments of $K_{E}^{D}$, $K_{E}^{I}$ and $Y_{E}$ are 0.14, 0 and 20671.83, respectively, when $m_{G}$ increases from 0.2 to 0.8), i.e. the dependency multiplier of enterprises' environmental governance gradually increases with the increase of government's marginal incentive propensity to enterprises, leading to a small increase in their environmental governance performance. Ultimately, in contexts where the enterprise's marginal governance propensity is low (e.g. $\beta_{E}=0.2$), the government–enterprise environmental performance decreases slowly as the government's marginal incentive propensity increases.

In a scenario with higher marginal environmental governance propensity of enterprises (e.g. $\beta_{E}=0.8$), it is clear from figure 3*b* that the pattern of the effect of government marginal incentive propensity on dependency multiplier, non-dependency multiplier, and respective environmental performance of government and enterprise does not change compared with the lower marginal environmental governance propensity scenario, but the magnitude of the change differs. Specifically, the increased values of $K_{G}^{D}$, $K_{G}^{I}$, $Y_{G}$, $K_{E}^{D}$, $K_{E}^{I}$ and *Y_E_* are −0.42, −0.66, −28959.30, 0.75, 0 and 72398.19, respectively, when $\beta_{E}=0.8$ and $m_{G}$ increases from 0.2 to 0.8; compared with the scenario with $\beta_{E}=0.2$, the above-mentioned increased value itself has changed by 70.72%, 100%, 87.56%, 525.36%, 100% and 350.23%.

It is more obvious that the magnitude of the change in the non-dependency multiplier remains the same in both types of subjects, but the increase in the enterprise governance dependency multiplier is higher than the decrease in the government environmental governance dependency multiplier. Similarly, the increase in environmental governance performance of the former is higher than the decrease in environmental governance performance of the latter. Therefore, the promotion effect on $Y_{E}$ due to the increase in the marginal incentive propensity of the government is greater than its inhibitory effect on $Y_{G}$ when the marginal environmental governance propensity of enterprise is high, which eventually leads to an increase in the government–enterprise environmental governance performance. Comparing figure 2, it is found that *Y* is always larger in the context of larger *β_E_* than in the context of smaller *β_E_* when $m_{G}$ is the same. It implies that the contribution of increased enterprise environmental governance propensity to government–enterprise environmental governance performance does not change essentially with changes in the level of marginal government incentives, but only affects the magnitude of the change.

**Figure 4.**
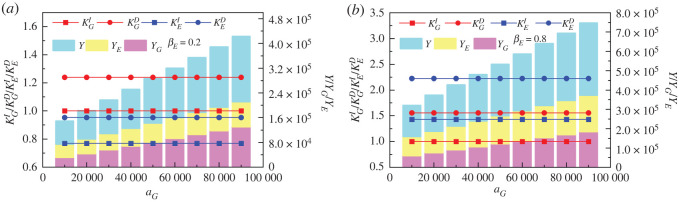
Influence of government environmental governance expenditure item (*a_G_*) on dependency multipliers ($K_{G}^{D}$ and $\;K_{E}^{D}$), non-dependency multipliers ($K_{G}^{I}$ and $\;K_{E}^{I}$) and environmental governance performance ($Y_{G}$, $\;Y_{E}$ and  Y). (*a*,*b*) correspond to the two scenarios with low ($\beta_{E}=0.2$) and high ($\beta_{E}=0.8$) marginal environmental governance propensity of enterprise, respectively. The red circles and squares stand for government dependency $\left(K_{G}^{D}\right)$ and non-dependency multipliers $\left(K_{G}^{I}\right)$. The blue circles and squares stand for enterprise dependency $\left(K_{E}^{D}\right)$ and non-dependency multipliers $\left(K_{E}^{I}\right)$. The light magenta, light yellow and light cyan bars stand for environmental governance performance of government, enterprise and the total, respectively. The other parameters are as follows: $m_{G}=m_{E}=0.5$, $\beta_{G}=0.5$, $a_{E}=40\;000$, K=0.1.

The fixed expenditure item of government environmental governance is the environmental governance cost in the annual environmental governance plan based on the local environmental characteristics and the macro-demand of national environmental governance. From figure 4*a*, it can be seen that *a_G_* increase has no effect on both dependent and non-dependent multipliers of government and enterprise, but can significantly change the environmental governance performance of both types of subjects, which in turn ultimately improves the overall environmental performance. Specifically, $Y_{G}=31428.57$, $Y_{E}=42857.14$ and Y=74285.71 when $a_{G}=10\;000$; $Y_{G}=118095.20$, $Y_{E}=76190.48$ and Y=194285.70 when $a_{G}=80\;000$. That is, $Y_{G}$, $Y_{E}$ and *Y* increased by *3.76* times, *1.78* times and *2.62* times, respectively.

As shown in figure 4*b*, in the higher enterprise environmental governance propensity scenario (*β_E_* = 0.8), $Y_{G}$, $Y_{E}$ and *Y* increase by 108888.90, 7777777.78 and 186666.70 for $a_{G}=10\;000$to $a_{G}=80\;000$, respectively, and the growth rate is 1.81, 0.78 and 1.17 times. Thus, the impact characteristics of government environmental governance fixed expenditure item on governance performance are not affected by changes in enterprise marginal governance propensity, but only change in the magnitude of the impact. This is mainly due to the fact that $K_{G}^{D}<K_{E}^{D}$ and $K_{G}^{I}<K_{E}^{I}$ in the scenario with $\beta_{E}=0.8$, and $K_{G}^{D}>K_{E}^{D}$ and $K_{G}^{I}>K_{E}^{I}$ in the scenario with $\beta_{E}=0.2$. i.e. both government and enterprise dependency multipliers and non-dependency multipliers increase as *β_E_* increases. However, the increase in the fixed expenditure item of government environmental governance contributes to the growth of dependency multiplier and non-dependency multiplier of government at a lower level than that of enterprise.

### 4.2. Dynamic evolution of public environmental governance behaviour

Individual environmental governance behaviours among the public are influenced by multiple factors, and their behavioural strategy transfer is a probabilistic learning mechanism influenced by the decision-making environment. Therefore, in order to reflect the influence of public behaviour on environmental governance performance more intuitively, the key influencing factors and their sensitivity characteristics of environmental collaborative governance are explored based on a systematic perspective.

**Figure 5.**
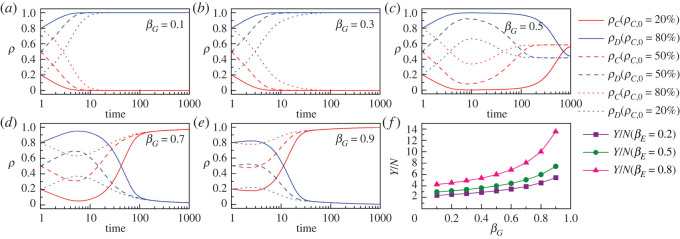
(*a–e*) present the evolutionary characteristics of public environmental governance cooperative behaviour ($\rho_{C}$) and defective behaviour ($\rho_{D}$) under different initial levels of cooperation ($\rho_{C,0}=20\%,\;50\%\;and\;80\%$) and government marginal environmental governance propensities ($\beta_{G}=0.1$, 0.3, 0.5, 0.7 and 0.9). The red and blue solid lines correspond to $\rho_{C,0}=20\%$; the red and blue dashed lines correspond to $\rho_{C,0}=50\%$; the red and blue dotted lines correspond to $\rho_{C,0}=80\%$. $\beta_{E}=0.5$ in (*a–e*). (*f*) presents the influence of government marginal environmental governance propensity on environmental governance performance *per capita* (Y/N) in scenarios where enterprises' marginal environmental governance propensity is low ($\beta_{E}=0.2$, purple squares line), moderate ($\beta_{E}=0.5$, olive circles line) and high ($\beta_{E}=0.8$, pink triangles line). The other parameters are as follows: $m_{G}=m_{E}=0.5$, $a_{G}=a_{E}=40\;000$, K=0.1.

At the overall level, with the introduction of public behaviour, the increase in the government marginal environmental governance propensity has a significant boosting effect on the *per capita* performance of government–enterprise environmental governance. As shown in figure 5*f*, *Y*/*N* increases gradually with increasing *β_E_*, and the increase in *β_G_* has an enhancing effect on the above boosting effect. Furthermore, how the micro-mechanism of public environmental governance behaviour behind the above facilitation effect works. Figure 5*a–e* reveals the effect of the government marginal environmental governance propensity on the evolution of environmental governance behaviour of social publics. Specifically, when *β_G_* = 0.1 and 0.3 (i.e. figure 5*a,b*), the number of social publics adopting cooperative behaviour in environmental governance activities decreases rapidly over time until it disappears completely, and the smaller *β_G_* the faster the cooperative behaviour disappears. When *β_G_* increases to a certain level (e.g. *β_G_* = 0.5 in figure 5*c*), cooperative behaviour of public environmental governance bounces back after an initial decline and reaches an evolutionary steady state at a certain level. Continuing to increase *β_G_* (e.g. *β_G_* is 0.7 and 0.9 in figure 5*d,e*, respectively), public cooperation experiences a brief decline before the emergence of all-Cs. By contrast, social public's defective behaviour first grows rapidly and then gradually declines. In general, an increase in the government environmental governance marginal propensity has positive implications for the improvement of the social public's environmental governance behaviour, i.e. when *β_G_* is small, it can slow down the decline of cooperative behaviour; when *β_G_* is moderate, it can make cooperative behaviour maintain at a certain level; when *β_G_* is large, it can make cooperative behaviour eventually tend to emerge completely. In addition, from figure 5*a–e*, it can be further found that the differences in the initial cooperative behaviour among social publics cannot fundamentally change the evolutionary properties. If the initial level of cooperative environmental governance behaviour in social publics is high, the level of cooperation is relatively high in the initial period of evolutionary process, and then the evolutionary stable state tends to be consistent as the evolutionary process goes further, which supports the importance of environmental policy guidance to some extent.

**Figure 6.**
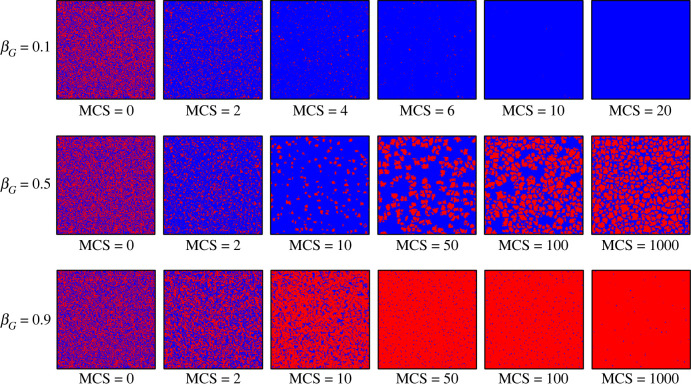
Snapshots of typical distributions of cooperators (red) and defectors (blue) guide by the government marginal environmental governance propensities ($\beta_{G}$) at different MCS. From the top to the bottom, $\;\beta_{G}=0.1$, 0.5 and 0.9, respectively, as well as all the results are obtained from $m_{G}=m_{E}=0.5$, $\beta_{E}=0.5$, $a_{G}=a_{E}=40\;000$, K=0.1.

The above macro-level analysis suggests that changes in the government environmental governance marginal propensity have an impact on both the evolutionary process and the evolutionary steady state of the public environmental governance behaviour. At the micro-level, figure 6 reveals the transformation characteristics of public environmental governance behaviours in the evolutionary process, where red indicates that publics adopt cooperative behaviour and blue indicates that the social public adopts defective behaviour, and the initial state is that the social public's environmental governance cooperative behaviour and defective behaviour are evenly dispersed in the social group. In scenarios where the government environmental governance marginal propensity is small (e.g. *β_G_* = 0.1), defective behaviour among the social public rapidly spreads to form larger clusters and tightly surrounds cooperative behaviour (Monte Carlo time (MCS) = 2, 4, 6). The cooperative behaviour affected by the peripheral defective behaviour still failed to resist the invasion of betrayal behaviour as the evolutionary process advanced (MCS = 10) and disappeared rapidly and completely (MCS = 20). In the context of a moderate government environmental governance marginal propensity (e.g. *β_G_* = 0.5), social public cooperative behaviour first decreases substantially and is surrounded by clusters formed by defective behaviour (MCS = 2). Cooperative behaviour then gradually formed clusters although still decreasing (MCS = 10), implying that the social public produced an influx of cooperative behaviour within a small number of communities in the environmental governance process. As the evolution progresses (MCS = 50, 100, 1000), the communities where this type of environmental governance cooperation behaviour emerges continue to spread and surround the clusters formed by defective behaviours, and finally the environmental governance cooperation behaviour in the group can be stabilized at a certain level. As evolution advances (MCS = 50, 100, 1000), the communities where environmental governance cooperative behaviour emerges are spreading and surrounding the clusters formed by the defective behaviour, which eventually enables the environmental governance cooperative behaviour in the group to stabilize at a certain level. In contexts where the government environmental governance marginal propensity is high (e.g. *β_G_* = 0.9), cooperative environmental governance behaviour among the public rapidly replaces defective behaviour and forms small cooperative clusters surrounding defection (MCS = 2). Subsequently, clusters of environmental governance cooperative behaviours spread to form larger cooperative behaviour emergent communities (MCS = 10). Finally, defective behaviours are replaced in large numbers and cooperative behaviours tend to emerge in social publics (MCS = 50, 100, 1000). Therefore, the increased propensity of government environmental governance helps to protect the emergence of cooperative environmental governance behaviours in small communities from being annihilated by defective behaviours.

**Figure 7.**
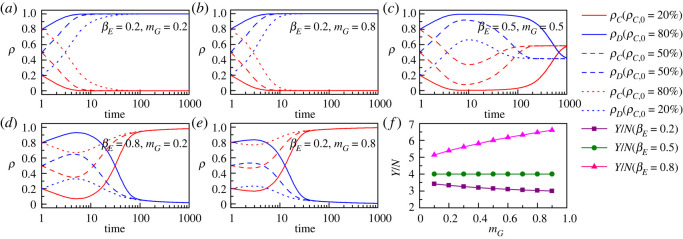
(*a–e*) present the evolutionary characteristics of public environmental governance cooperative behaviour ($\rho_{C}$) and defective behaviour ($\rho_{D}$) under different initial levels of cooperation ($\rho_{C,0}=20\%,\;50\%\;and\;80\%$) and government marginal incentive propensity (*m_G_* = 0.2 and 0.8). The red and blue solid lines correspond to $\rho_{C,0}=20\%$; the red and blue dashed lines correspond to $\rho_{C,0}=50\%$; the red and blue dotted lines correspond to $\rho_{C,0}=80\%$. $\beta_{E}=0.2,\;0.5$ and 0.8 in (*a–e*), respectively. (*f*) presents the influence of government marginal incentive propensity (*m_G_*) on environmental governance performance *per capita* (*Y*/*N*) in scenarios where enterprises' marginal environmental governance propensity is low ($\beta_{E}=0.2$, purple squares line), moderate ($\beta_{E}=0.5$, olive circles line) and high ($\beta_{E}=0.8$, pink triangles line). The other parameters are as follows: $m_{E}=0.5$, $\beta_{G}=0.5$, $a_{G}=a_{E}=40\;000$, K=0.1.

It can be seen from figure 7 that the influence of government's marginal incentive propensity (*m_G_*) on social publics' environmental governance behaviour is regulated by the enterprise environmental governance marginal propensity (*β_E_*). Specifically, in the scenario where *β_E_* is small (e.g. *β_E_* is 0.2 in figure 7*a,b*), when *m_G_* is raised from 0.2 to 0.8, the cooperative behaviour of social publics disappears rapidly as the evolutionary process advances (MCS = 10), and defective behaviour always emerges as an evolutionary equilibrium. Evolutionary stable state of social publics’ environmental governance behaviour is not affected by the initial level of cooperation, and defective behaviour can still quickly replace cooperative behaviour in the context of a higher initial level of cooperation (e.g. $\rho_{C,I}=80\%$). Similarly, in contexts with moderate values of *β_E_* (e.g. *β_E_* is 0.5 in figure 7*c*) and large values of *β_E_* (e.g. *β_E_* is 0.8 in figure 7*d–e*), the public environmental governance evolutionary steady state cannot be changed by simply increasing the government's marginal incentive propensity.

It is worth noting that the characteristics of the effect of *m_G_* on the *per capita* level of government–enterprise environmental governance performance (*Y*/*N*) depend on *β_E_*. Specifically, in figure 7*f*, as *m_G_* increases, *Y/N* decreases, does not change and increases at $\beta_{E}=0.2$, $\beta_{E}=0.5$ and $\beta_{E}=0.8$, respectively. The above effects will have a moderating effect on the evolution of social publics' environmental governance behaviour. For example, as shown in figure 7*a,b*, *m_G_* increases from 0.2 to 0.8 at an initial cooperation level of 0.2, and the evolutionary steady state emerges earlier from MCS = 18 to MCS = 11. *m_G_* increases from 0.2 to 0.8 at an initial cooperation level of 0.8, and the evolutionary steady state emerges earlier from MCS = 137 to MCS = 45.

**Figure 8.**
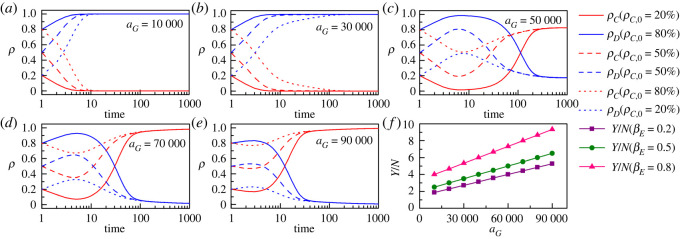
(*a–e*) present the evolutionary characteristics of public environmental governance cooperative behaviour (*ρ_C_*) and defective behaviour (*ρ_D_*) under different initial levels of cooperation ($\rho_{C,0}=20\%,\;50\%\;and\;80\%$) and government environmental governance fixed expenditure items ($a_{G}=10\;000$, 30 000, 50 000, 70 000 and 90 000). The red and blue solid lines correspond to $\rho_{C,0}=20\%$; the red and blue dashed lines correspond to $\rho_{C,0}=50\%$; the red and blue dotted lines correspond to $\rho_{C,0}=80\%$. $\beta_{E}=0.5$ in (*a–e*). (*f*) presents the influence of government environmental governance fixed expenditures on environmental governance performance *per capita* (*Y*/*N*) in scenarios where enterprises' marginal environmental governance propensity is low ($\beta_{E}=0.2$, purple squares line), moderate ($\beta_{E}=0.5$, olive circles line) and high ($\beta_{E}=0.8$, pink triangles line). The other parameters are as follows: $m_{G}=m_{E}=0.5$, $\;\beta_{G}=0.5$, $a_{E}=40\;000$, *K* = 0.1.

Moreover, figure 8 reveals the impact of changes in government environmental governance fixed expenditures on the evolutionary characteristics of social publics’ environmental governance behaviour. Specifically, when *a_G_* is small (e.g. *a_G_* is 10 000 and 30 000 in figure 8*a,b*, respectively), the behaviour of the social public rapidly shifts to full defective behaviour (MCS *<* 10) and is maintained in that evolutionary steady state. When *a_G_* increases to a certain value (e.g. $a_{G}=50\;000$ in figure 8*c*), the evolutionary characteristics of the social public's environmental governance behaviour change, and the proportion of cooperative behaviour first decreases rapidly, then gradually increases and maintains at a high level. Further increasing the value of *a_G_* (e.g. *a_G_* is 70 000 and 90 000 in figure 8*d,e*, respectively), it is found that the social publics' cooperative behaviour can become an evolutionary stable state. Also, as presented in figure 8*f*, the improvement of fixed expenditures items of government environmental governance is conducive to the improvement of *per capita* environmental governance performance and is positively motivated by the marginal environmental governance propensity of enterprises. In summary, under the condition that other conditions remain unchanged, the cooperative behaviour of social publics can be better improved by increasing the government environmental governance fixed expenditure item, and the above findings are not affected by the initial level of cooperative behaviour. Even in a situation where the initial level of cooperation is low (e.g. the initial cooperation level is 0.2), the emergence of cooperative behaviour can still be achieved by increasing the government environmental governance fixed expenditure item.

## 5. Discussion

Environmental collaborative governance is essentially a game of interests among multiple subjects, requiring all parties involved to reach a positive-sum game relationship of mutual restraint and mutual promotion in interaction. This paper explores evolutionary dynamics of public behaviour in environmental collaborative governance based on a two-stage model. Based on the simulating results performed in this study, some conclusions can be obtained. First, a higher marginal propensity for environmental governance helps to improve the interdependence between the government and enterprises, thereby promoting the public to take cooperative actions to participate in environmental governance. The government can increase the dependency multiplier and non-dependency multiplier of its environmental governance by increasing the marginal governance propensity, thereby creating environmental governance spillover benefits to improve environmental performance. At the same time, the gradual increase of the government's marginal governance propensity can indirectly reduce the decay rate of public cooperative behaviour and improve the ability of cooperative behaviour groups to resist the invasion of defective behaviour and achieve full emergence. Therefore, the government should increase the marginal governance propensity as much as possible within the resource-constrained space and increase the improvement of social and public environmental governance behaviour. In addition, the initial level difference of public environmental governance cooperation tends to be consistent as the evolution progresses to a stable state, which to a certain extent proves the importance of government environmental policy.

Second, the government's marginal incentive propensity to enterprise environmental governance indirectly affects the public's environmental governance behaviour and is regulated by the enterprise marginal governance propensity. The increase of the government's marginal incentive propensity to enterprises can improve the performance of corporate environmental governance. However, when the marginal governance propensity of enterprises is low, the decline rate of government environmental performance is faster than the growth rate of enterprise environmental performance, which is not conducive to environmental performance and the emergence of public cooperation. However, when the marginal environmental governance propensity of enterprises is high, increasing the marginal incentive propensity of the government to enterprises is conducive to the improvement of environmental governance performance and promotes the emergence of public environmental governance cooperation. Therefore, only raising the level of environmental governance incentives cannot guarantee efficient environmental governance performance and public behaviour, and comprehensive measures should be taken to improve the marginal environmental governance propensity of enterprises.

Third, the fixed expenditure items of government environmental governance are the environmental governance costs determined in the annual environmental governance plan according to the local environmental characteristics and the macro needs of national environmental governance. Although the increase of fixed government expenditure items cannot significantly affect the environmental governance dependency and non-dependency multipliers of the government and enterprises, it can improve the environmental governance performance and promote the public to adopt cooperative behaviours in environmental governance. And the above conclusions are not affected by the public's initial level of cooperative behaviour. Even if the initial level of cooperation is low, it is still possible to improve public environmental governance behaviour by increasing government environmental governance fixed expenditure items.

However, some limitations of this study should be noted. First, the focus of this study is on how the government can improve the cooperative behaviour of public participation in environmental governance, and the issues of the risk of political and commercial complicity in source governance, or relatively obvious non-cooperation under the government-business environmental governance framework are not considered. With the in-depth penetration of digital technology and information technology in the field of environmental governance [76], public participation in environmental governance can supplement information, constrain power, solve the information and power asymmetry between the government and enterprises, and play a role as a bond between multiple subjects in environmental governance. Thus, the mechanisms underlying digital and communication technology-driven public participation in collaborative environmental governance will be a focus of our future attention. Second, the different impacts of public participation behaviour on environmental performance [15,77] are not distinguished comprehensively in our two-stage evolutionary dynamics model. However, in practice, there are great differences in the role positioning, social interaction and information channels of the public in specific environmental governance scenarios. There are multiple heterogeneities in public behaviour, and their impact on environmental performance is also quite different. Future research plans to further divide the public into different types of subjects from the perspective of the environmental governance platform and study differentiated incentive measures in a targeted manner.

## Data Availability

The data are provided in the electronic supplementary material [78].
